# Influenza Treatment: Limitations of Antiviral Therapy and Advantages of Drug Combination Therapy

**DOI:** 10.3390/microorganisms11010183

**Published:** 2023-01-11

**Authors:** Sania Batool, Santosh Chokkakula, Min-Suk Song

**Affiliations:** Department of Microbiology, Chungbuk National University, College of Medicine and Medical Research Institute, Cheongju 28644, Chungbuk, Republic of Korea

**Keywords:** influenza virus, antiviral drugs, antiviral drug resistance, drug combination therapy

## Abstract

Influenza infection is serious and debilitating for humans and animals. The influenza virus undergoes incessant mutation, segment recombination, and genome reassortment. As a result, new epidemics and pandemics are expected to emerge, making the elimination challenging of the disease. Antiviral therapy has been used for the treatment of influenza since the development of amantadine in the 1960s; however, its use is hampered by the emergence of novel strains and the development of drug resistance. Thus, combinational therapy with two or more antivirals or immunomodulators with different modes of action is the optimal strategy for the effective treatment of influenza infection. In this review, we describe current options for combination therapy, their performance, and constraints imposed by resistance, calling attention to the advantages of combination therapy against severe influenza infections. We also discuss the challenges of influenza therapy and the limitations of approved antiviral drugs.

## 1. Introduction

“Flu” is the colloquial term used to describe an emerging disease whose major causative agent is influenza virus A or B. It is a contagious infection that spreads from one person to another through patient sneezes or coughs. Influenza symptoms include a runny nose, dry cough, sore throat, high fever, chills, headaches, and body aches [1]. In most cases, patients improve within a few days; however, when complications develop, flu can also cause morbidity and mortality, resulting in epidemics.

Mode of Action of Influenza Virus Replication: Such as all viruses, influenza viruses require invading a host cell to replicate, release, and subsequently spread. This process involves several stages.

Attachment and Entry: The influenza virus enters the host through the attachment of its hemagglutinin (HA), a type of glycoprotein present in the viral envelope, to sialic acid residues on the glycoprotein or glycolipid receptors of the host. The cell then endocytoses the virus, after which the HA protein undergoes a change in shape and unites with the endosomal membrane within the acidic environment of the endosome [2]. Viral ribonucleoprotein (vRNP) complexes are then released into the host cytoplasm and travel to the nucleus of the host cell, where they replicate and generate viral mRNA transcripts. This viral entry step can be an attractive target for viral inhibition strategies that block the ion channel and disable the release of vRNP into the cytoplasm [3].

RNA and Protein Production: Viral vRNP complexes carry out primary transcription in the host’s nucleus, producing the proteins needed for replication. The mechanism of primary transcription is called “cap snatching,” in which the 5′ methylguanosine cap of the mRNA of the host is bound by polymerase protein 2 (PB2), followed by removal of the cap and 10–13 nucleotides by the viral endonuclease, PA. The resulting oligonucleotide is used by a viral polymerase protein 1 (PB1) as a primer for transcription. This stage of viral processing is also a potential target for antiviral therapy. By inhibiting the cap-snatching process, the transcription of viral mRNA can be blocked, thereby inhibiting the production of the proteins necessary for virion formation [4]. The ten primary proteins produced by the translation of the eight segments of the genome in influenza A and B include one of the components of the vRNP complex, two matrix proteins (M1 and M2), two NS proteins (NS1 and NEP), neuraminidase (NA), PB1, PB2, nucleoprotein (NP), and HA. RNA-dependent RNA polymerase can be selectively inhibited by antiviral agents, making this another possible target for viral inhibition [5].

Assembly and Release: Following the creation of the initial proteins, the eight negative-sense viral RNA (vRNA) segments in influenza A and B are used to create eight positive-sense complementary RNA (cRNA) strands. These strands are missing the 5′ capped primer and the 3′ poly (A) tail found in mRNA. A negative sense of vRNA is created from this cRNA. Various viral proteins generated subsequently assemble and assist this vRNA in exiting the nucleus and entering the cytoplasm of the host cell [6].

During this period, HA and NA are glycosylated, polymerized, and acylated in the cytoplasm and, together with M2, are directed to the plasma membrane, where they become anchored. The proteins then interact with the other matrix protein, M1, and start the budding process. The virus buds after at least eight RNA segments arrive at the location via a yet unknown mechanism. Finally, NA cleaves the sialic acid of the HA protein of the progenitor, allowing the virus to exit the cell. This NA-mediated step is an attractive inhibitory target as preventing the breakdown of sialic acid residues would reduce viral infectivity by restricting progeny virus dispersal inside mucosal secretions [7].

## 2. Classes of Approved Antivirals for Influenza

So far, four classes of antivirals, including adamantanes, neuraminidase inhibitors, RNA-dependent RNA polymerase inhibitors, and polymerase acidic endonuclease inhibitors, are considered the approved class of antivirals to treat influenza patients. The detailed information on each class of these antivirals is systematically deliberated below.

### 2.1. Adamantanes—M2 Ion Channel Blockers

A detailed description of the approved antiviral drugs and their mode of action is summarized in Table 1. Adamantanes, also known as M2 ion channel blockers, limit the early stages of virus replication by blocking the ion channel formed from the M2 protein encoded by the M gene of the influenza A virus [8]. If treatment is initiated within 48 h of infection, it has the potential to shorten the illness by 1.5 days [9]. Because of their low cost and ready availability, adamantanes have been used for almost 50 years to treat infections caused by the influenza A virus [10]. This class consists of two drugs,

(i) Amantadine—the first drug developed for treating influenza infections; approved by the US Food and Drug Administration (FDA) in 1966.
(ii) Rimantadine—an α-methyl derivative of amantadine (α-methyl-1-adamantane methylamine hydrochloride) approved by the FDA in 1993.

Neither of these drugs is currently used in the US owing to high antiviral resistance.

**Table 1:** Approved classes of antivirals for influenza virus.

Drug	Class	Target Action	Brand Name	Virus Type	Administration Route	Approval Agency	Approval Year
Amanadine	M2 ion channel blockers	Blocks the viral uncoating and entry into the host cell	Symmetrel	A	Oral	No longer	1963
Rimantadine	M2 ion channel blockers	Blocks the viral uncoating and entry into the host cell	Flumadine	A	Oral	No longer	1969
Zanamivir	NA inhibitor	Blocks viral NA activity	Relenza	A & B	Nasal or Oral	FDA	1993
Oseltamivir	NA inhibitor	Blocks viral NA activity	Tamiflu	A & B	Oral	FDA	1998
Peramivir	NA inhibitor	Blocks viral NA activity	Rapivab, Peramiflu	A & B	I.M, I. V	FDA	2000
Laninamivir	NA inhibitor	Blocks viral NA activity	Inavir	A & B	Nasal	Japan	2010
Favipiravir	RNA polymerase inhibitor	Inhibits viral RNA-dependent RNA polymerase substrate	Avigan	A & B	Oral	Japan, China	2002
Baloxavir marboxil	Polymerase acidic Endonuclease inhibitor	Inhibits the activity of cap-dependent endonuclease	Xofluza	A & B	Oral	FDA	2018

FDA—Food and Drug Administration, I.M—Intramuscular, I.V—Intravenous

### 2.2. Neuraminidase Inhibitors

Until 2018, neuraminidase inhibitors (NAIs) represented the only FDA-approved class of antivirals. The BM2 protein in influenza B viruses is such as the M2 protein in influenza A viruses; however, it is not susceptible to M2 blockers. Moreover, most circulating human influenza viruses are resistant to M2 blockers; therefore, the World Health Organization (WHO) does not recommend their use. Despite these caveats, NAIs are approved for use against both influenza A and B infections and do stop viruses from spreading and infecting healthy cells [11]. There are four globally licensed NAIs for prophylaxis and treatment of influenza virus infections:

(i) Zanamivir (tradename, Relenza)—the first drug of the NAI class [12], approved by the FDA in 1999. Zanamivir is a potential inhibitor of NA, a surface glycoprotein that possesses enzymatic activity critical for the replication of influenza A and B viruses. Owing to its poor oral bioavailability, zanamivir can either be administered intranasally or by oral inhalation. The recommended dosing of zanamivir for the treatment of influenza A or B is 10 mg by oral inhalation twice daily for 5 days. Treatment should begin as soon as possible after the onset of symptoms and no later than 48 h after the first appearance of symptoms [13]. Early zanamivir treatment in individuals with spontaneously acquired influenza is linked to a shorter duration of symptoms, lower use of antibiotics, and a quicker return to normal activity levels [14].
(ii) Oseltamivir (trade name, Tamiflu)—another NAI that suppresses influenza NA enzymes necessary for virus replication. An orally administered agent currently available as oral capsules as well as a suspension formulation [8], oseltamivir is used for 5 days at two doses per day for therapeutic purposes and once daily for up to 42 days for prophylaxis [15]. Oseltamivir is taken in the form of oseltamivir phosphate, an oral prodrug that is easily converted to the active form, oseltamivir carboxylate [16]. All influenza viruses have NA enzymes, which are necessary for the release of progeny virions from infected host cells in affected individuals. Oseltamivir carboxylate attaches to and inhibits the active sites of these enzymes [17]. By preventing viral replication, oseltamivir carboxylate reduces viral load and shortens the duration of infection. In addition to minimizing the intensity and length of influenza symptoms, if administered within 48 h of becoming ill, it can decrease the possibility of complications such as pneumonia, otitis media, and bronchitis [17]. Prophylactic administration can also help to prevent symptomatic illnesses [17]. Oseltamivir, studied *in vitro*, shows activity against all NA subtypes, including those of human seasonal viruses, avian viruses [18], and pandemic viruses, such as the recently emerged pandemic (H1N1) 2009 (A(H1N1) pdm09) virus [19], reflecting the fact that the active site of the NA enzyme is extremely conserved [18].
(iii) Peramivir (trade name, Rapivab)—accepted for use in 2010 in Japan and Korea and approved by the FDA in 2014 for the treatment of acute, uncomplicated influenza. Before the introduction of peramivir, which was developed as an intravenous (IV) antiviral agent, there was no NAIs available for intravenous delivery to patients in critical condition [20]. Peramivir binds to the NA enzyme more effectively than other NAIs, inhibiting the *in vitro* activity of influenza A and B virus [20,21].
(iv) Laninamivir (trade name, Inavir)—a long-acting NAI [22] first approved for use in 2010 in Japan that is still undergoing clinical trials in other countries. Administered by nasal inhalation in the form of the prodrug laninamivir octanoate, laninamivir is hydrolyzed in the lungs, where it is maintained at a high concentration in its active form for a considerable amount of time, thereby effectively inhibiting influenza viral replication. Therapeutic plasma concentrations of laninamivir can persist for up to 144 h following inhalation; thus, only a single inhalation is required to treat influenza [23].

### 2.3. RNA-Dependent RNA Polymerase Inhibitor

This class of antivirals, which consists of the single drug favipiravir, inhibits viral RNA synthesis. Though still not approved by the FDA, it was approved in Japan in 2014 for the treatment of novel and re-emerging influenza virus infections and in China in 2016. Favipiravir is more effective than oseltamivir in treating influenza infections [24,25]. A notable attribute of favipiravir is the apparent lack of development of favipiravir-resistant viruses, a testament to the quality of favipiravir as an antiviral drug.

As a chain terminator, favipiravir prevents viral RNA production. This contrasts with drugs that inhibit genomic RNA synthesis, which leads to the production of drug-resistant mutations by viral RdRp, a component of the low-fidelity RdRp-viral RNA complex. This is the most important feature that distinguishes favipiravir from prior anti-influenza drugs [26]. Other anti-influenza medications, such as NAIs and baloxavir (see below), prevent the transmission of infection but facilitate the production of genomic RNA, which can drive the creation of drug-resistant viruses.

### 2.4. Polymerase Acidic Endonuclease Inhibitor

This is a new class of antivirals consisting of the single drug baloxavir marboxil (trade name, Xofluza), which was first approved in Japan in 2018, and later the same year by the FDA for treating uncomplicated flu. A single oral dose of baloxavir marboxil is required to treat influenza.

Baloxavir marboxil is a specific inhibitor of the cap-dependent endonuclease of the viral RdRp complex. In the presence of baloxavir, the integrated RdRp-viral RNA complex synthesizes cRNA without the cap structure, which limits mRNA synthesis and causes failure of subsequent viral protein synthesis.

In phase 3 clinical trials, baloxavir marboxil significantly decreased the time to remission influenza symptoms compared with a placebo. Additionally, it reduced the infectious viral titer and duration of virus shedding more rapidly than oseltamivir in both high-risk individuals and patients who were otherwise healthy [27,28]. However, this most recently developed anti-influenza medication is only accessible in a few countries and has unknown efficacy in clinical settings.

## 3. Challenges of Influenza Therapy

Because of its ability to change and its capacity to resist even the most powerful immune responses, the influenza virus remains a constant threat to its hosts while continuing to survive in populations to which it has previously been exposed. Antigenic shifts and drifts in the influenza virus’s surface glycoproteins are two primary pathways for the antigenic evolution of the virus. Both mechanisms, which have evolved to overcome natural immunity, make vaccination difficult.

The major challenge in treating influenza is the antiviral resistance of most human influenza A virus strains toward M2 inhibitors. Adamantanes work only against influenza A viruses and are not effective against influenza B viruses. An adamantane-resistant influenza A virus was identified during a 1980 outbreak [29], but until 2000 only 1–2% of seasonal influenza A subtypes showed resistance to both amantadine and rimantadine [30,31]. This percentage has increased substantially since then [32], such that, by 2013, approximately 45% of all extant influenza A virus subtypes worldwide showed resistance to adamantanes [33] (Table 2). A high prevalence of adamantane-resistant influenza A(H3N2) and A(H1N1) pdm09 viruses has been observed in recent seasons. As a result, amantadine and rimantadine antiviral therapy and chemoprophylaxis are not suggested for use against presently circulating influenza A viruses [34].

Another shortcoming in treating influenza virus infections is the requirement for *in vivo* disease models for influenza-related research, which can be challenging and problematic from the standpoint of evaluating antiviral treatment and efficacy. Numerous animal models often require repeated analysis of host-virus interactions, transmission methods, and host immunological reactions to various influenza viruses. Antivirals that target influenza can be evaluated using a variety of animal models, but each has its advantages and disadvantages. Mice are the most popular model for *in vivo* investigations, especially for preclinical evaluations of influenza antivirals. That said, ferrets are best suited as an animal model for studying novel antiviral drugs because these animals can contract a variety of human, avian, and swine influenza strains and recapitulate many clinical symptoms observed in humans after viral infections [35]. However, because of their complex husbandry requirements and high cost, ferret models are rarely used in research. Guinea pigs, on the other hand, lack characteristic clinical symptoms and therefore are rarely employed to test antiviral drugs [35]. Cotton rats are viewed as a replacement for the mouse model owing to their small size and less complicated husbandry needs. One study reported that, after infection with the influenza A virus, antiviral therapy with either zanamivir or oseltamivir reduced pathology scores and lung lesions in this animal model [36]. Nevertheless, this animal model is not often utilized to study anti-influenza drugs. Non-human primates are also used as animal models, with some studies showing that oseltamivir, zanamivir, and peramivir are effective at lowering the viral load of influenza and A and B viruses in these models [37,38,39,40]. However, such primate models come with even more complicated husbandry conditions than the ferret model and are expensive.

Until 2018, drug combination therapy for influenza was limited to only two classes of drugs, but after the approval of a new class of drugs by the FDA, researchers developed an interest in exploring how the efficacy of antivirals used in combination therapy is impacted by their mechanism of action. However, testing a large range of dosages for combination therapy in a research setting is costly and time-consuming. The combined effect of the drugs may be greater or less than the sum of their individual effects due to drug interactions, phenomena referred to as “synergy” and “antagony,” respectively [41]. Drug interactions can take place at any point in the drug’s life cycle, including absorption, excretion, and metabolism. Drug-drug interactions must be carefully studied since adverse interactions between drugs can result in undesired outcomes [42].

## 4. Limitations of Antivirals and Anti-Influenza Drug-Resistance Mechanisms

Apart from these challenges, the emergence of drug-resistant influenza strains due to currently prescribed antiviral drug regimens is a major limitation in influenza treatment, and such related significant limitations of influenza monotherapy are detailed below.

### 4.1. Adamantanes

As noted above, adamantanes were the first approved antiviral class for the treatment of influenza. Although they are effective against influenza A (but not influenza B), they are not currently recommended for the treatment of influenza A owing to increased levels of resistance [43]. M2 inhibitor-resistant strains can spread from person to person, are pathogenic, and can occasionally be recovered from untreated individuals. Human isolates of very virulent A/H5N1 influenza viruses are naturally resistant to these antivirals [44].

Approximately 95% of adamantane-resistant influenza A virus subtypes have been discovered to harbor an S31N mutation [33,45]. This mutation causes adamantane resistance in 98.2% of H3N2 isolates, with the remainder (1.8%) attributable to L26F, V27A, and A30T mutations [32]. The swine-origin influenza A H1N1 subtype that triggered the pandemic of 2009 also had an S31N mutation in M2 that made it resistant to amantadine and rimantadine [46]. Similarly, the avian influenza A virus subtypes H5N1 and H7N9, which originated in 2003 and 2013, respectively, and caused serious zoonotic infections in humans, both have the S31N mutation in M2 and are therefore adamantane resistant [47] (Table 2).

### 4.2. NAIs

As is the case with M2, multiple amino acids in or surrounding the active site of NA in the influenza A virus have changed during the development of resistance to NAIs [48], including E119V, R292K, I222V, H274Y, and N294S. Most of these mutations directly or indirectly change the shape of the active site of NA, drastically reducing NAI binding [49,50,51].

Although resistance to M2 inhibitors became more prevalent in influenza A virus H3N2 subtypes, resistance to NAIs initially emerged and became predominant in the H1N1 subtype [45]. Oseltamivir, an antiviral that comes in capsule or powdered form for the preparation of liquid suspensions, has a high oral bioavailability. Zanamivir, on the other hand, is a less suitable NAI because it can only be given through inhalation [17]. Because of oseltamivir’s widespread use since 2000, influenza variants harboring NA mutations H274Y, N294S, Y252H, I223R/V, and some others have developed resistance to the drug [52,53,54,55].

Surprisingly, at that time, some oseltamivir-resistant strains were sensitive to zanamivir [56]. H1N1 and H5N1 subtypes harboring the oseltamivir (and peramivir) resistance-causing H274Y mutation in NA were the most prevalent strains, but they were still susceptible to zanamivir. E119V and R292K mutations are more common in H3N2 and H7N9 subtypes [57]. In contrast, the Q136K mutation in NA caused H1N1 and H3N2 isolates to become resistant to zanamivir or make them less susceptible to it [57]. Studies conducted in 2009–2012 demonstrated the prevalence of peramivir resistance in 1.3–3.2% of influenza A/H1N1pdm09 viruses but in less than 1% of influenza A/H3N2 and B viruses [58,59,60]. The I221T, A245T, K360E, A395E, and D432G mutations and the G145R + Y142H combined mutation are all found in a single variant [58]. Some reverse genetics-generated viruses that carry known NA mutations (e.g., E119G, E119V, and Q136K) in an A(H1N1) pdm09 virus and confer reduced to severely reduced laninamivir inhibition have been described [11,60,61,62], but to date, no laninamivir-resistant mutations have been identified in clinical samples [11,63]. Previous research has suggested that laninamivir, either by itself or in combination with interferon lambda 1 (IFN-λ1), promotes the formation of the E119G NA mutation in Calu-3 cells, an effect that was associated with considerably reduced susceptibility to laninamivir and zanamivir [64]. Three HA mutations—D127E, T197A, and D222G—were found to be present in all variants transmitted in the presence of laninamivir alone and in combination with IFN-λ1 [64] (Table 2).

Some other mutations have been linked to decreased susceptibility to NAIs, but these mutations are not quite common.

### 4.3. Favipiravir

Only people treated with favipiravir can develop acquired resistance to the drug [59]. However, an investigation showed that an early isolate from the 2009 H1N1 pandemic known as influenza A/England/195/2009 (Eng195) might exhibit favipiravir resistance [65]. This study reported that two mutations, K229R in the motif F of PB1 and P653L in PA, were required for the evolution of resistance to favipiravir. K229R conferred resistance to favipiravir at the expense of polymerase activity, an effect that was balanced by the P653L mutation [65] (Table 2).

Although the use of favipiravir is unlikely to be common enough in the community to support selection for resistance, pre-existing drug-resistant mutations have spread in the absence of widespread drug use [66]. Favipiravir resistance could grow across the community through drift or through combination with other beneficial mutations if there is no cost to resistance.

Favipiravir is currently recommended only in China and Japan. Apart from emerging resistance, favipiravir’s poor solubility in aqueous media is another limitation that may reduce its effectiveness *in vitro* [67]. Furthermore, favipiravir was found to raise the risk of embryotoxicity and teratogenicity, indicating the need for strict regulation of its production and medical application. Additionally, although favipiravir does not possess the typical properties of nucleoside analogs that might cause mitochondrial toxicity, harmful effects on mitochondria cannot be fully ruled out [68,69].

### 4.4. Baloxavir Marboxil

Influenza A viruses have been shown to develop resistance to baloxavir through mutations at isoleucine 38 (I38X), a highly conserved residue in the PA catalytic site (Table 2). According to a study by Omoto and colleagues, mutations in the influenza virus’s PA protein reduce the susceptibility to baloxavir [70]. Specifically, influenza strains carrying E23/G/K, E36V, E37T, I38T/F/M/L, E119D, or E119G mutations in PA are significantly more resistant to baloxavir in vitro [71,72,73]. Among these, PA I38T is a major mutation that reduces baloxavir susceptibility and alters its EC_50_ values for influenza A by 30- to 50-fold and for influenza B by 7-fold [70].

**Table 2:** Reports of drug mutations and their frequencies in different influenza virus types.

Influenza Virus Type	Drug	Assay	No. of Tested Isolates	Mutation Percentage	Mutation Detected	Ref.
A & B	Zanamivir	NAI, S	41	0	-	[74]
B	Oseltamivir	NAI, S	77	0	1 Gly402Ser	[75]
A & B	Oseltamivir	NAI, S	759 A, 256 B	2.5% (A-2.5%, 0%-B)	2 Arg292Lys, 17 His275Tyr	[76]
A	Oseltamivir	Cloning + S	50	18%	1 Asn294Ser,2 Glu119Val, 6 Arg292Lys	[77]
A	Oseltamivir	NAI, S	54	4%	2 His274 Tyr	[78]
A	Oseltamivir	PCR	30	15.6%	4 His275Tyr	[79]
A & B	Oseltamivir	NAI, S	43 A, 19 B	1.3% (A-1.3%, 0%-B)	1Arg292Lys, 3 His275Tyr	[80]
A	Oseltamivir	NAI, S	418	1%	1 Glu119Val,4 Arg292Lys	[81]
B	Oseltamivir	NAI, S	74	16%	7 His274Tyr	[82]

NAI—Neuraminidase Inhibition Assay, S—Sensitivity, and PCR—Polymerase Chain Reaction.

## 5. Drug Combination Therapy: As an Alternative Treatment Option for the Influenza Infection

**Figure 1:**
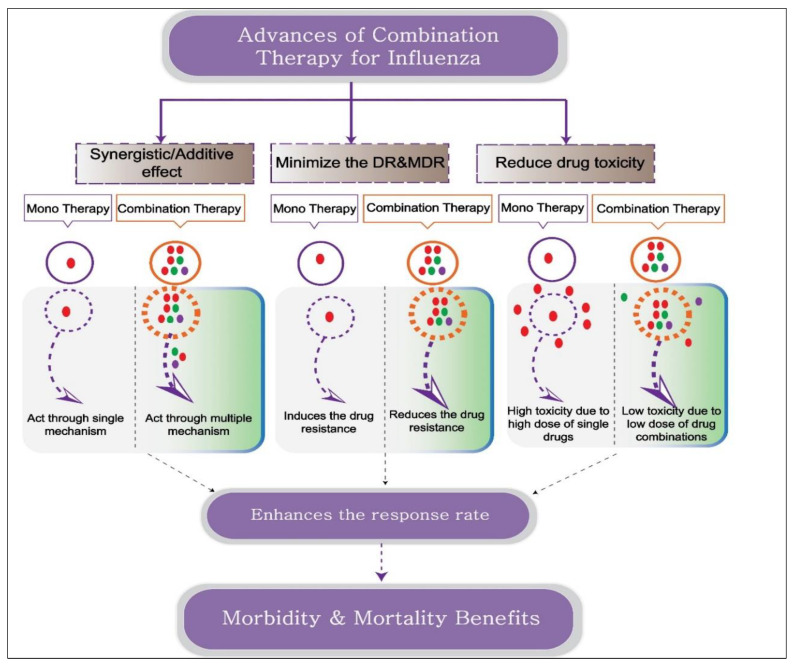
Schematic representation depicting the advantages of combination therapy over monotherapy. Combination therapy includes two or more drugs or immunomodulators with the same or different therapeutic targets. Antiviral combination with different therapeutic activities promotes synergistic action, reducing drug toxicity and drug resistance proportion. The combination of an antiviral and an immune modulator targets virus and host factors, thereby inhibiting virus replication and simultaneously enhancing the host defense mechanism and conferring morbidity and mortality benefits.

Combination drug therapy is a therapeutic strategy in which a disease is treated with two or more drugs. To promote drug synergism, researchers can design combination therapy to target multiple pathways of the host and pathogen, thereby resulting in benefits greater than simple additive effects (Figure 1). In addition to enhancing therapeutic efficacy, this can enable drugs to be used at lower, individualized dosages, thereby increasing patient tolerance and reducing drug toxicity. The likelihood of resistance to two antivirals is decreased compared with that to a single antiviral, so combination therapy serves to increase the antiviral spectrum. The main goal of combination therapy, which is typically given to critically ill patients when the first-line therapy fails, is to eliminate the infection and restore normal functioning (Figure 1). This is already the gold standard approach for treating several viral infections, including HIV-1 and HCV. Although the development of novel drugs through traditional pipelines remains essential, rapid identification of effective drug candidates through drug-repurposing screens is an ideal method for accelerating potential emergency use. It is considerable *in vitro* and *in vivo* data on combination antiviral therapy that support the value of combination therapy over monotherapy. In addition, several phase I-, II-, and III-stage clinical studies are currently underway that offer hope of producing combinations with demonstrated value in the treatment of acute human influenza infections. Some of the many antivirals and immunomodulator combination options that have shown increased antiviral efficacy in patients with serious influenza infections, including those who are also immunocompromised and thus carry an increased risk of severe disease and emergence of resistance, are detailed below. Antiviral or immune modulator components of combination therapy that target the same or different viral proteins or host factors are also described here in detail. Various *in vitro*, *in vivo*, and clinical studies reporting the advantage of combination therapy, such as single-target combination therapy, multi-target combination therapy, and host and pathogen target combination therapy), are described in detail below.

### 5.1. Antiviral Combination Therapy Targeting Same Viral Protein

Combination therapy with two different drugs belonging to the same class is possible if the drugs possess completely different binding interactions with the viral protein. The oseltamivir + zanamivir and oseltamivir + peramivir combinations belong to this category (Table 3).

Oseltamivir + zanamivir: Oseltamivir and zanamivir are both members of the NAI class, but they bind to different NA active sites. In a mixed infection model, oseltamivir-zanamivir combination therapy suppressed oseltamivir and zanamivir-resistant viruses very effectively [83]. This approach might assist in maintaining the clinical value of NAIs by preventing the growth of drug-resistant strains. A clinical study demonstrated a reduction in viral load and improved clinical response in individuals treated with oseltamivir and zanamivir combination therapy compared with oseltamivir alone [NCT00830323]. In addition to its clinical significance, this combination therapy also prevents secondary transmission of the disease [NCT00799760].

**Figure 2:**
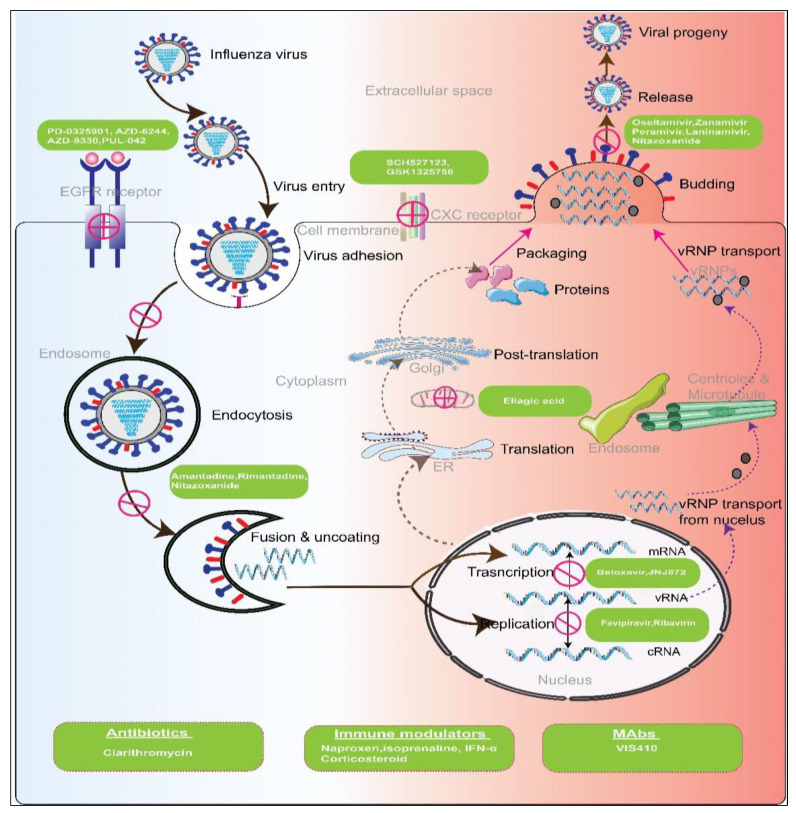
Potential targets of antiviral and other immunomodulators for different factors of pathogen and host. Influenza virus enters the cytoplasm by receptor-mediated endocytosis by recognition of sialic acid receptors. The low pH in the endosome induces the release of RNA, a therapeutic target of several antivirals. Transcription and replication from RNA occur in the nucleus, and both steps are inhibited by antivirals targeting the PA endonuclease and RdRps. Packaging and release of the virion occur at the cell membrane, and neuraminidase inhibitors block this step. Immunomodulators and other agents target host factors and stimulate the immune system against viral infection.

Oseltamivir + peramivir: Oseltamivir plus peramivir is another combination whose individual components target the same viral mechanism. Although both compounds act as neuraminidase inhibitors, peramivir binds more strongly to the enzyme than oseltamivir and thus confers protection against the influenza A virus. Twice daily administration of these drugs in influenza-infected mice was shown to significantly increase survival rate compared with a single therapy, demonstrating that combination therapy with these two drugs has an advantage in treating influenza compared with a suboptimal dose of the single drug [84] (Figure 2).

### 5.2. Antiviral Combination Therapy Targeting Two or More Viral Proteins

Drugs in combination therapy target two or different pathways of the influenza virus is an effective treatment strategy. It improves therapeutic efficacy by providing synergistic activity, lower drug dose utility, and better treatment response rate. There are several different drug combinations against influenza therapy, which are listed below (Table 4).

#### 5.2.1. Antiviral Combination Therapy Targeting Cap-Dependent Endonuclease & RNA-Dependent RNA Polymerase

Antiviral combination therapy targeting cap-dependent endonuclease & RNA-dependent RNA polymerase has shown its advantage over both drugs used alone (Table 4).

Baloxavir + favipiravir: A recent *in vitro* investigation of combination therapy reported synergistic effects of baloxavir-favipiravir combination therapy [85]. However, considerable additional investigation will be required to confirm the effectiveness of combination therapy with these two polymerase inhibitors against resistant influenza strains.

#### 5.2.2. Antiviral Combination Therapy Targeting Cap-Dependent Endonuclease & Neuraminidase

The synergistic effects of antivirals targeting the cap-dependent endonuclease & neuraminidase of the influenza virus have been reported in several studies (Table 4).

Baloxavir + oseltamivir: Although findings from a recent study suggest that baloxavir and NAI combination therapy is not generally advisable for treating acute influenza patients in a clinical setting [86], some studies have reported synergistic effects of baloxavir-oseltamivir combination therapy in mice [87,88]. Compared with monotherapy, a suboptimal dose of the antiviral drug baloxavir marboxil together with oseltamivir phosphate showed improved efficacy in terms of virus-induced mortality, cytokine/chemokine production, and morphological changes in the lungs [88]. However, this study did not provide any evidence of the effectiveness of combined therapy against resistant influenza strains. Subsequent research by Park et al. in 2021 supports the combined use of baloxavir and oseltamivir to effectively combat influenza while still reducing the occurrence of antiviral drug resistance [88]. This combination therapy recently entered two clinical studies, one on the treatment of allogeneic bone marrow transplant recipients with influenza [NCT05170009] and the other on immunocompromised patients with severe influenza infection [NCT05170009]. The findings of these clinical studies could establish the broad-spectrum antiviral efficacy of this combination with sufficient rigor.

**Table 4:** Antivirals combination therapy target two or more proteins of the influenza virus.

Drug Combinations	Target	Virus	Efficacy Evaluating Model	Clinical Advantage/Primary Outcome Measures	Ref.	Status	Clinicaltrials.gov ID
Oseltamivir + JNJ-63623872	Neuraminidase& Cap-dependent endonuclease	A	Clinical	Reduce the influenza load	-	II	NCT03040141
Baloxavir + Favipiravir	Cap-dependent endonuclease & RdRp	A	*In vitro*	Improved antiviral activity	[85]	-	-
Baloxavir + Oseltamivir	Cap-dependent endonuclease & Neuraminidase	A	*In vivo*	Against Baloxavir, PA-resistant influenza	[87,88]	-	-
Baloxavir + Oseltamivir		A	Clinical	Recruiting	-	II, III	NCT05170009
Baloxavir + Oseltamivir		A	Clinical	Recruiting	-	II	NCT04712539
Favipiravir + Oseltamivir	RdRp & neuraminidase	A & B	Clinical	Faster clinical improvement	[89]	-	-
Peramivir +Favipiravir		A	*In vivo*	Against the oseltamivir-resistant influenza virus infection	[90,91]	-	-
Peramivir + Rimantadine	M2 ion channel & neuraminidase	A	*In vivo*	Synergistic antiviral effects.	[92]	-	-
Oseltamivir + Rimantadine		A	*In vivo*	Improved morbidity and mortality	[93]	-	-
Oseltamivir + Amantadine		A	*In vivo*	Provided protection	[94]		
Oseltamivir + Amantadine		A	Clinical	Appears safe	[95]	I	NCT00416962
Oseltamivir + Amantadine		A	Clinical	Terminated	-	II	NCT00830323
Oseltamivir +Amantadine + Ribavirin	M2 ion channel, neuraminidase & RdRp	A	*In vivo*	Synergistic activity	[96,97,98]		
Oseltamivir + Amantadine + Ribavirin		A	*In vivo*	Reduce mortality and weight loss	[99]	-	-
Oseltamivir + Amantadine + Ribavirin		A	Clinical	Effective in virus clearance	-	II	NCT00979251
Oseltamivir + Amantadine + Ribavirin		A	Clinical	significant decrease in viral shedding	[100]	II	NCT01227967
Oseltamivir + Amantadine + Ribavirin		A	Clinical	Reduce the influenza load	[101]	I, II	NCT00867139

#### 5.2.3. Antiviral Combination Therapy Targeting RNA-Dependent RNA Polymerase & Neuraminidase

Another class of antivirals targeting the RdRp, and neuraminidase of influenza virus have demonstrated their therapeutic utility in several in vitro, in vivo, and clinical studies (Table 4).

Favipiravir + oseltamivir: In a mouse model, this combination offered *in vivo* synergy, providing 60–80% protection together with improved body weight maintenance during A(H1N1) and A(H3N2) influenza virus infection [89]. A recent study performed a similar comparative analysis in humans to assess the efficacy of oseltamivir plus favipiravir combination therapy and oseltamivir monotherapy [90]. Although the sample size used for evaluating favipiravir-oseltamivir combination therapy was small compared with that of oseltamivir monotherapy, combination therapy was nevertheless demonstrated to hasten patient recovery from influenza infection [90]. These results indicate that, in individuals with chronic influenza, combining favipiravir and oseltamivir may improve clinical outcomes and increase therapeutic efficacy.

Peramivir + favipiravir: Favipiravir is regarded as an essential component of combination therapy for the treatment of both seasonal and pandemic influenza. A study examining combination therapy in DBA/2 mice using peramivir and favipiravir as an antiviral-resistant treatment showed that infected mice were protected from severe viral pathogenesis by the synergistic action of these two agents [76]. In humans, this paired approach has demonstrated therapeutic effectiveness against naturally occurring oseltamivir-resistant H1N1 strains [91].

#### 5.2.4. Antiviral Combination Therapy Targeting the M2 Ion Channel & Neuraminidase

Various influenza mutations resulting from the widespread use of M2 ion channel blockers, together with adjunctive antivirals such as neuraminidase inhibitors, have provided superior protection against influenza infection as combination therapy.

Peramivir + rimantadine: Combined administration of the neuraminidase inhibitor, peramivir, and M2 ion channel blocker, rimantadine, for five consecutive days was shown to cause significant and synergistic weight loss in mice infected with influenza A virus [96]. These antivirals target two distinct viral proteins—an advantage compared with targeting a single protein. Therefore, this study design may form the basis for further clinical studies to verify the efficacy of this combination in the treatment of patients infected with influenza.

Oseltamivir + rimantadine: An evaluation of prophylactic and therapeutic benefits of the drug combination, oseltamivir plus rimantadine, investigated two different dose patterns in a mouse model. This combination showed a protective index of ~79% in a prophylactic setting and ~80% during a therapeutic course [93] and effectively reduced viral titer, pneumonia parameters, and individual drug dosage requirements. This study clearly established that the combination of oseltamivir and rimantadine exerts a greater protective effect compared with monotherapy in both prophylactic and therapeutic contexts in mice.

Oseltamivir + amantadine: Combination therapy in mice using doses of oseltamivir and amantadine corresponding to those used in humans was shown to result in 90% protection against lethal infection with an amantadine-sensitive H5N1 virus. Viral shedding to the brain was also effectively controlled by this combination, and no mutations in HA, NA, or M2 protein were reported [94]. This combination therapy provided a survival advantage over monotherapy, making it the best option for treating influenza. A randomized, crossover study evaluating the pharmacokinetics of the amantadine and oseltamivir combination reported no evidence of an increase in adverse events [95]. More clinical studies are needed to determine the adverse events, drug-drug interactions, and pharmacokinetic consequences associated with this combination.

#### 5.2.5. Antiviral Combination Therapy Targeting the M2 Ion Channel, Neuraminidase & RNA-Dependent RNA Polymerase

In addition to the dual target antiviral combinations listed above, antiviral combination therapy with more than two viral targets, such as the M2 ion channels, neuraminidase & RNA-dependent RNA polymerase, is more advantageous against drug-resistant strains of influenza (Table 4).

Oseltamivir + amantadine + ribavirin: Triple-combination antiviral drug treatment (TCAD) with amantadine, oseltamivir, and ribavirin has shown synergistic activity *in vitro* against both drug-sensitive and drug-resistant strains of influenza A [96,97]. Compared with dual combination therapy, TCAD exerts a greater inhibitory effect and is also more effective in reducing the occurrence of resistance during *in vitro* passage [98]. In an *in vivo* model, TCAD therapy was reported to be highly effective in reducing mortality and weight loss in mice infected with the influenza A virus. It was also shown to provide survival support in cases where treatment is delayed (up to 72 h) after viral infection [99]. In addition, three important clinical trials are currently ongoing in patients with different disease manifestations. A phase 2 open-label, the randomized study reported that TCAD is safer in clearing viral shedding compared with oseltamivir monotherapy for the treatment of all strains of influenza A in immunocompromised and pediatric subjects [NCT00979251]. Another randomized, double-blind, multicenter phase 2 trial of adults infected with influenza A H1N1, A H3N2, or B virus suggested that the TCAD combination significantly improved viral clearance relative to oseltamivir monotherapy [100]. A safety and pharmacokinetics efficacy study with healthy and immune-compromised influenza patients also demonstrated evidence of the superior safety of this drug combination [101]. Additional follow-up studies are needed, as this approach seems to have potential benefits in fighting antiviral resistance by targeting more features of the influenza virus.

M2 blockers such as amantadine and rimantadine are no longer recommended to treat Influenza patients due to the increased prevalence of drug resistance strains across the globe beginning during 2003–2004. Therefore, the use of the M2 blockers as a combination therapy may limit the synergistic efficacy of influenza treatment.

### 5.3. Combination Therapy Targeting Host and Pathogen Molecular Mechanisms

In addition to direct antiviral combinations, other host-targeted therapeutics, such as monoclonal antibodies, immunomodulators, cell signaling inhibitors, anti-inflammatory agents, and antioxidant agents, can be co-administered together with direct antiviral agents as part of a combination therapy approach for treating drug resistance, immunocompromised patients, and patients with severe influenza (Figure 2) (Table 5). Such approaches offer superior advantages compared with single-drug treatment (Figure 1).

Because a neuraminidase inhibitor (e.g., oseltamivir) alone is less effective in treating patients with severe influenza, adjunctive therapy with the antibiotic, clarithromycin, and anti-inflammatory drug, naproxen, in a combination regimen provides effective protection, reducing mortality, hospital stay, and defervescence in adult influenza patients. This combination therapy mounted a more rapid antiviral defense and produced a more rapid decline in viral load in hospitalized pediatric influenza patients [102,103]. The Raf/MEK/ERK signaling pathway plays a central role in the replication of the influenza virus. Hence, MEK inhibitors such as PD-0325901, AZD-6244, AZD-8330, and RDEA-119, together with oseltamivir, show increased antiviral activity *in vitro*, laying the groundwork for the development of an alternative therapeutic regimen against influenza through further *in vivo* and clinical studies [104]. The neutrophil chemokine receptor, CXCR2 (CXC chemokine receptor 2), is the main determinant of neutrophil infiltration and a therapeutic target for reducing inflammation. The combination of oseltamivir and the CXCR2 antagonist, SCH527123, was shown to markedly enhance survival in mice and attenuate the severity of lung pathology in infected animals [105]. Danirixin (GSK1325756), another CXCR2 antagonist, effectively engages its target to elevate IL-8 levels and produce a suitable clinical response with no serious side effects when co-administered with oseltamivir [NCT02927431] [106]. Ligands of the toll-like receptors TLR2, −6, and 9 are host-targeted immune stimulants that control the progression of several diseases, including influenza. Treatment of mice with the TLR ligand PUL-042, together with oseltamivir, was shown to provide superior prophylactic and therapeutic control of infection and improve survival rate [107]. Systemic corticosteroid therapy is usually preferred for the treatment of acute respiratory distress syndrome, but combining this corticosteroid with oseltamivir was shown to further improve the prognosis of chronic lung disease patients [108]. Combinations that act on three different therapeutic targets could offer additional advantages. The neuraminidase inhibitor, oseltamivir, immune modulator, isoprenaline, and antioxidant agent, ellagic acid, administered together in influenza-infected mice were shown to provide such an advantage, improving the protection index and increasing survival time while reducing lung titer, lung pathology, and superoxide radicals [109]. The investigational drug, JNJ872, targets the viral protein PB2 and halts the transcription of viral genes associated with virus replication. A phase 2 study evaluating the antiviral efficacy, pharmacokinetics profile, and safety of the combination of JNJ-63623872 and oseltamivir reported significant clinical improvement in adult and elderly hospitalized influenza patients [NCT02532283]. VIS410, an HA stem-targeted monoclonal antibody (mAb-IgG1), was shown to elicit a better clinical outcome, eliminate the need for oxygen support, and reduce viral load when given together with oseltamivir [NCT03040141]. The role of interferons (IFNs) in the antiviral innate immune defense against influenza is well established. A pilot phase 2 clinical study in adult influenza patients revealed that the combination of IFN-α and oseltamivir produced an effective clinical response, resolving fever and flu symptoms [NCT01146535]. An *in vitro* analysis has shown that nitazoxanide, an anti-parasite drug with broad antiviral activity, including against influenza, synergizes with oseltamivir [110,111]. Prophylactic administration of the combination of oseltamivir and nitazoxanide in a ferret model resulted in significantly less virus shedding and complete elimination of the virus from the lower respiratory tract [111]. A recently completed phase III clinical trial [NCT01610245] of oseltamivir plus nitazoxanide combination treatment demonstrated the resolution of all clinical symptoms of influenza as the primary outcome (Table 5).

## 6. Conclusions

Several therapeutic strategies are currently being tested for treating influenza, but these efforts are challenged by rising antiviral resistance, a problem that will require some critical actions to resolve. First, the use of antivirals should be limited, as it is necessary to preserve the limited influenza drug options for both seasonal and pandemic influenza. The compulsory nature of this constrained use of antivirals should be impressed on those responsible for patient treatment. Secondly, more research on anti-influenza agents is necessary, as these drugs remain relatively understudied. (For example, compared with research on anti-HIV drugs, which garnered 13,024 items in PubMed in the last 5 years, only 337 items match “anti-influenza drugs” during the same period.) Third, research on *in vitro* and clinical testing approaches for pairing agents for combination therapy should be expanded, given the challenges associated with *in vivo* testing. Finally, there should be an increased focus on developing new antiviral treatment strategies, such as exploring effective combination therapies, to improve the use of existing antivirals and mitigate the emergence of resistance.

## Figures and Tables

**Table 3:** Antiviral combination therapy targets the same proteins of the influenza virus.

Drug Combinations	Target	Virus	Efficacy Evaluating Model	Clinical Advantage/Primary Outcome Measures	Ref.	Status	Clinicaltrials.gov ID
Oseltamivir + Zanamivir	Neuraminidase	A	*In vitro*	Against the NAI-resistant influenza strains	[83]	-	-
Oseltamivir+ Peramivir		A	*In vitro*, *in vivo*	Synergistic antiviral effects	[84]	-	-
Oseltamivir + Zanamivir		A	Clinical	Terminated	-	II	NCT00830323
Oseltamivir + Zanamivir		A	Clinical	Terminated- Results not available	-	III	NCT00799760

**Table 5:** Combination therapy targets both host and pathogen proteins.

Drug Combinations	Target	Virus	Efficacy Evaluating Model	Clinical Advantage/Primary Outcome Measures	Ref.	Status	Clinicaltrials.gov ID
Oseltamivir + Clarithromycin + Naproxen	Neuraminidase, 23S rRNA & cyclooxygenase	A & B	Clinical	Faster clinical improvement	[102,103]	-	-
Oseltamivir + MEK inhibitors	Neuraminidase & Raf/MEK/ERK signaling pathway	A	*In vitro*	Synergistic antiviral effect	[104]	-	-
Oseltamivir + CXCR2 antagonist	Neuraminidase & CXCR2	A	*In vivo*	Improved morbidity and mortality	[105]	-	-
Oseltamivir + Danirixin	Neuraminidase & CXCR2	A	Clinical	Proper clinical response	[106]	II	NCT02927431
Oseltamivir +TLR- 2,6 9 ligand	Neuraminidase & TLRs	A	*In vivo*	Improved protection	[107]	-	-
Oseltamivir + Corticosteroid	Neuraminidase & inflammatory signaling	B	Clinical	Better prognosis	[108]	-	-
Oseltamivir + Isoprinosine + Ellagic acid	Neuraminidase, RNA synthesis & tyrosinase	A	*In vivo*	Protective effects	[109]	-	-
Oseltamivir + VIS410	Neuraminidase & HA stem	A	Clinical	Reduce the influenza load	-	II	NCT03040141
Oseltamivir + Interferon-alpha	Neuraminidase & IFN-α	A	Clinical	Resolution of the illness	-	II	NCT01146535
Oseltamivir + Nitazoxanide, Nitazoxanide	Neuraminidase & M2 ion channel	A	*In vitro*, *In vivo*	Synergistic antiviral effects	[110,111]	-	-
Oseltamivir + Nitazoxanide	Neuraminidase & M2 ion channel	A	Clinical	Reduction in the clinical symptoms	-	III	NCT01610245
